# Dynamic Evolution of Vascular Features Based on Magnetic Resonance Imaging to Predict Pathological Response, Patterns of Recurrence and Survival Outcomes in Breast Cancer Neoadjuvant Chemotherapy

**DOI:** 10.3390/curroncol32060350

**Published:** 2025-06-13

**Authors:** Qiong Wu, Mingxi Zhu, Huaying Xie, Xiaochuan Geng, Yan Wang, Ziping Wu, Yanping Lin, Shuguang Xu, Yumei Ye, Wenjin Yin, Zhiguo Zhuang, Jingsong Lu, Liheng Zhou

**Affiliations:** 1 Department of Breast Surgery, Renji Hospital, School of Medicine, Shanghai Jiao Tong University, Shanghai 200127, China; wqlucy3790@163.com (Q.W.); aureliane@163.com (M.Z.); 20220@renji.com (Y.W.); wuziping@renji.com (Z.W.); linyanping@renji.com (Y.L.); xushuguang@renji.com (S.X.); yeyumei@renji.com (Y.Y.); yinwenjin@renji.com (W.Y.); 2 Department of Radiation Oncology, Renji Hospital, School of Medicine, Shanghai Jiao Tong University, Shanghai 200127, China; xiehuaying@renji.com; 3 Department of Radiology, Renji Hospital, School of Medicine, Shanghai Jiao Tong University, Shanghai 200127, China; gengxiaochuan@renji.com (X.G.); zhuangzhiguo@renji.com (Z.Z.); 4 Punan Branch of Renji Hospital, School of Medicine, Shanghai Jiao Tong University (Punan Hospital in Pudong New District, Shanghai), Shanghai 200127, China

**Keywords:** breast cancer, neoadjuvant chemotherapy, magnetic resonance imaging, vascular feature, pathological response, survival

## Abstract

Magnetic resonance imaging (MRI) is essential for assessing responses to neoadjuvant chemotherapy (NAC) in breast cancer. This exploratory analysis included 182 patients with locally advanced breast cancer who underwent MRI at baseline after two NAC cycles and post-treatment. Vessel-through-lesion (VTL) was quantified as the number of vessels passing through the lesion, with ΔVTL_1-2_ and ΔVTL_1-3_ representing relative VTL changes after the second and third MRI, respectively, compared to baseline. Patients were randomly divided into training and validation sets (7:3). A bpCR-predictive nomogram incorporating ΔVTL_1-2_ and clinicopathological factors out-performed a clinicopathology-only model (validation AUC of 0.892 vs. 0.857). Recurrence patterns differed by ΔVTL_1-3_ subgroup, where the low ΔVTL_1-3_ group showed an early recurrence peak within two years, while the high ΔVTL_1-3_ group maintained a stable, low recurrence risk over five years, particularly among non-pCR patients. The RFS-predictive nomogram using ΔVTL_1-3_ showed strong concordance with RFS (validation AUCs of 0.918 at 1 year, 0.767 at 3 years, and 0.717 at 5 years). Risk stratification showed superior RFS in the low-risk group. This study is the first to report that dynamic vascular evolution predicts bpCR and serves as an independent prognostic factor, associated with distinct recurrence patterns, particularly in non-pCR patients. These findings support early identification of patients for intensified post-NAC adjuvant therapy, enabling more precise treatment.

## Simple Summary

Neoadjuvant chemotherapy (NAC) is commonly used to reduce tumor burden in breast cancer prior to surgery. However, accurate prediction of therapeutic response remains a clinical challenge. In this study, we used breast MRI scans to analyze the number of blood vessels passing through the tumor at different stages of treatment (baseline, after two cycles of NAC and before surgery). Our results showed that patients with a greater reduction in tumor-associated blood vessels were more likely to achieve breast pathological complete response and longer relapse-free survival. Moreover, distinct vascular change patterns were correlated with different recurrence types. These findings suggest that MRI-derived vascular features may serve as non-invasive imaging biomarkers for predicting treatment efficacy and recurrence patterns, potentially guiding personalized therapeutic strategies for intensified post-NAC adjuvant therapy.

## 1. Introduction

Neoadjuvant chemotherapy (NAC), followed by surgery and adjuvant therapy, constitutes the prevailing therapeutic paradigm for patients with locally advanced breast cancer (LABC). NAC facilitates tumor downstaging, thereby augmenting the prospects for breast-conserving surgery and diminishing the exigency for superfluous axillary lymph node dissections [1]. It has been elucidated that pathological complete response (pCR) post-NAC is indicative of enhanced survival outcomes, particularly in cases of human epidermal growth factor receptor-2 (HER2) positive/hormone receptor (HR) negative and triple negative pathologies [2]. A substantial cohort of patients continues to confront the peril of suboptimal tumor response. Consequently, investigative endeavors are imperative to segregate individuals with an elevated likelihood of pCR and to estimate the potential prognosis, thereby identifying potential predictive determinants for personalized tumor responses and survival.

Magnetic resonance imaging (MRI) of the breast is a widely recognized modality for the diagnosis and assessment of breast cancer. In patients undergoing NAC, initial breast MRI serves as a viable technique for delineating the extent of the lesion and assessing nodal status. While previous MRI-based paradigms have shown commendable efficacy in forecasting pCR and survival outcomes [3,4,5], they are encumbered by substantial constraints, such as a paucity of patient data, reliance on singular subtype-specific characteristic, and esoteric feature extraction methodologies. Thus, there is an imperative for further investigations to devise more universally accessible and precise models to anticipate tumor response to NAC and subsequent survival metrics. Contrast-enhanced MRI offers a means to assess tumor vascularity using contrast agent injection [6]. The identification of the vascular map obtained by MIP could offer a novel perspective on tumor characteristics. Studies have shown that unilateral elevation in breast vascularity is linked to ipsilateral invasive breast cancers [7,8,9]. In the neoadjuvant setting, vascular features might serve as predictors of tumor response to NAC [10]. Yet, the predictive value of the dynamic changes in vascular features for tumor response and survival remain unexplored.

In this study, we postulate that vascular features, in conjunction with clinicopathological characteristics, could facilitate precocious prediction of tumor responses and survival, as well as discriminating temporal recurrence patterns of breast cancer patients. We endeavor to quantify the dynamic evolution of vascular features discerned from MRI throughout NAC.

## 2. Materials and Methods

### 2.1. Patients and Treatment

We performed an exploratory analysis on breast cancer patients retrospectively enrolled from the prospective neoadjuvant database of Renji Hospital, School of Medicine, Shanghai Jiao Tong University. Eligible criteria included female patients with histologically confirmed primary unilateral breast cancer (T1 N1–3 M0 or T2–4 N0–3 M0) who received NAC and surgical treatment. Patients were excluded if they were pregnant, had metastatic breast cancer, bilateral breast cancer, had a previous history of malignancy other than breast cancer or did not complete the full process of NAC. Briefly, the therapeutic regimen comprised weekly 80 mg/m^2^ paclitaxel for 16 weeks, coupled with cisplatin on days 1, 8, and 15, out of every 28 days for 4 cycles. For HER2 positive disease, concurrent trastuzumab was administered as anti-HER2 target therapy on a weekly basis (first dose of trastuzumab was 4 mg/kg of body weight and the subsequent doses were 2 mg/kg). To evaluate the response to NAC, MRI assessments were conducted before NAC (baseline), subsequent to 2 NAC cycles (early treatment), and prior to surgical intervention post-NAC (preoperative). Following NAC, patients proceeded to receive further surgical treatment. After surgery, patients continued to undergo subsequent therapy at the discretion of investigators or according to the guidelines at that time. Specifically, HER2-positive patients continued to receive adjuvant trastuzumab after surgery for a total duration of one year. Between August 2014 and December 2018, a cohort of 182 patients, each having undergone both pre-NAC (baseline) and post-NAC MRI scans, was assembled for analysis. Among these patients, data of the second MRI scan, performed after two cycles of NAC, were available for 157 individuals.

### 2.2. MRI Protocols

MR imaging was conducted using a 3-T system (Ingenia, Philips Medical Systems, Best, The Netherlands) with patients in the prone position and both breasts suspended in a dedicated bilateral, four-channel, phased-array breast coil. Dynamic contrast-enhanced (DCE) imaging was performed with a T1-weighted high resolution isotropic volume examination (THRIVE) sequence after transverse diffusion-weighted imaging (DWI). Postprocessing involved image subtraction (contrast-enhanced minus unenhanced) and maximum intensity projection (MIP). Axial MIPs were obtained to visualize the whole vascular maps of both breasts, and vessel measurements were manually performed by an experienced breast surgeon blinded to the histopathologic data.

### 2.3. MRI Vascular Feature Extraction

Patients received three MRI scans, respectively, labeled as the first, second, and third MRI. We define Vessel-through-lesion (VTL) as the number of vessels crossing the lesion on the MIP image. VTLs of the three MRIs of a patient were measured at the same level under unified parameters, denoted as VTL_1_, VTL_2_, and VTL_3_. We subsequently define ΔVTL_1-2_ as the relative change in VTL after 2 cycles of NAC, calculated by

(1) $${\Delta VTL}_{1\text{-}2}=\frac{{VTL}_{1}-{VTL}_{2}}{{VTL}_{1}}\times 100\%$$

Similarly, ΔVTL_1-3_ is defined as the relative change in VTL completion of NAC, i.e.,

(2) $${\Delta VTL}_{1\text{-}3}=\frac{{VTL}_{1}-{VTL}_{3}}{{VTL}_{1}}\times 100\%$$

For both ΔVTL_1-2_ and ΔVTL_1-3_, a positive value indicates decreased number of vessels through lesion after NAC. The optimal cutoff of ΔVTL_1-2_ and ΔVTL_1-3_ were calculated (surv_cutpoint function; survminer package in R) to classify patients into high-ΔVTL_1-2_, low-ΔVTL_1-2_, high-ΔVTL_1-3_, and low-ΔVTL_1-3_ groups.

### 2.4. Data Collection

At baseline, clinical data including age, menopausal status, body mass index (BMI), clinical T stage, and clinical N stage were collected. Pathologic data from biopsy tissues, including histologic grade, estrogen receptor (ER) status, progesterone receptor (PR) status, HER2 status, and Ki-67 status, were obtained from Department of Pathology, Renji Hospital, Shanghai Jiaotong University School of Medicine. Hormone receptor (HR) positivity was defined as ER and/or PR ≥ 1% of nuclei stained using standard immunohistochemistry (IHC) methods. The cutoff value of Ki-67 was 50% [11,12]. The molecular types were defined according to the St. Gallen International Expert Consensus [13].

### 2.5. Outcomes

The outcomes were breast pathological complete response (bpCR), total pathological complete response (tpCR), relapse-free survival (RFS), disease-free survival (DFS), and overall survival (OS). The definition of bpCR is having no invasive cancer in the breast (ypT0/Tis). tpCR indicates the absence of any invasive cancer in the breast and lymph nodes (ypT0/Tis ypN0). RFS is the time from surgery to the first occurrence of locoregional, ipsilateral, contralateral, distant recurrence, or death from any cause. DFS is the time from surgery to the first occurrence of locoregional, ipsilateral, contralateral, distant recurrence, second primary cancer, or death from any cause. OS is the time from surgery to death from any cause.

### 2.6. Model Development and Validation

All the eligible patients were randomly assigned to the training set and validation set in a 7:3 ratio. The least absolute shrinkage and selection operator (LASSO) algorithm and 10-fold cross validation were used for selection of the parameters, according to which nomograms were constructed to individualize the predictive models for bpCR and RFS. Receiver operating characteristic (ROC) analysis was performed and the area under the curve (AUC) was calculated to assess the accuracy. The calibration curve was plotted to compare the consistency between actual outcomes and the predicted probabilities. The clinical utility of the models was evaluated using decision curve analysis (DCA).

### 2.7. Statistics

The chi-square test was used to compare categorical variables, and the Student’s *t* test was used to compare continuous variables. RFS, DFS, and OS were calculated using the Kaplan–Meier analysis and log-rank test. Logistic regression analyses were performed to calculate the odds ratios (ORs) and 95% confidence intervals (CIs). Cox proportional hazard regression was established to derive hazard ratios with 95% CIs. ΔVTL_1-2_-related calculations were performed only in the 157 patients with available MRI data after two NAC cycles. No imputation was used for missing imaging data. The individual risk scores calculated by the RFS prediction nomogram in the training set were used to obtain the optimal cutoffs (100 points) to provide the largest discrepancy in RFS, DFS, and OS (surv_cutpoint function; survminer package in R). The results were considered significant with *p* < 0.05. All analyses were performed by R software version 4.3.1 (http://www.R-project.org).

## 3. Results

### 3.1. Patient Clinicopathologic Characteristics

**Figure 1:**
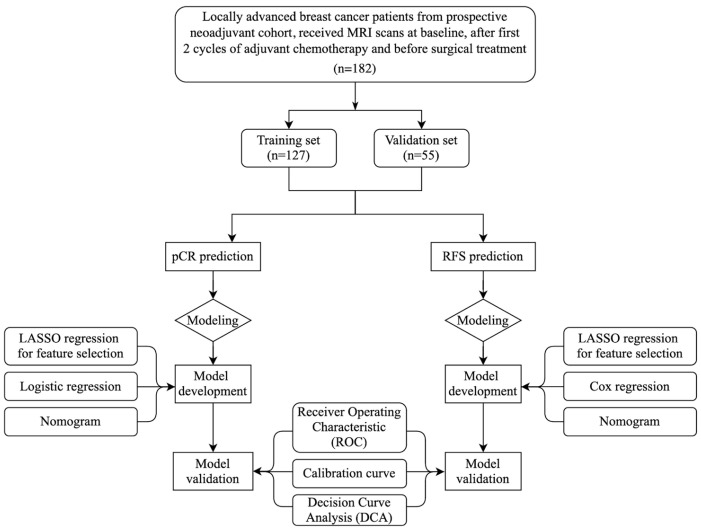
Flowchart of study design.

A flowchart of the study design is shown in Figure 1. Clinicopathological characteristics, vascular features, and survival data are well balanced between training set and validation set. Among 182 patients, 72 (39.56%) achieved bpCR (Table 1).

As categorical variables, 66 (42.04%) patients had high ΔVTL_1-2_ and 150 (82.42%) patients had high ΔVTL_1-3_ (Table 1). By investigating the relationship between vascular features and clinicopathological characteristics, we found that HR negativity (adjusted *p* = 0.045) and histologic grade-3 (adjusted *p* = 0.045) were associated with high ΔVTL_1-2_, and histologic grade-3 (adjusted *p* = 0.010) were associated with high ΔVTL_1-3_ (Table 2, Supplementary Tables S1 and S2).

### 3.2. Tumor Response to NAC

**Figure 2:**
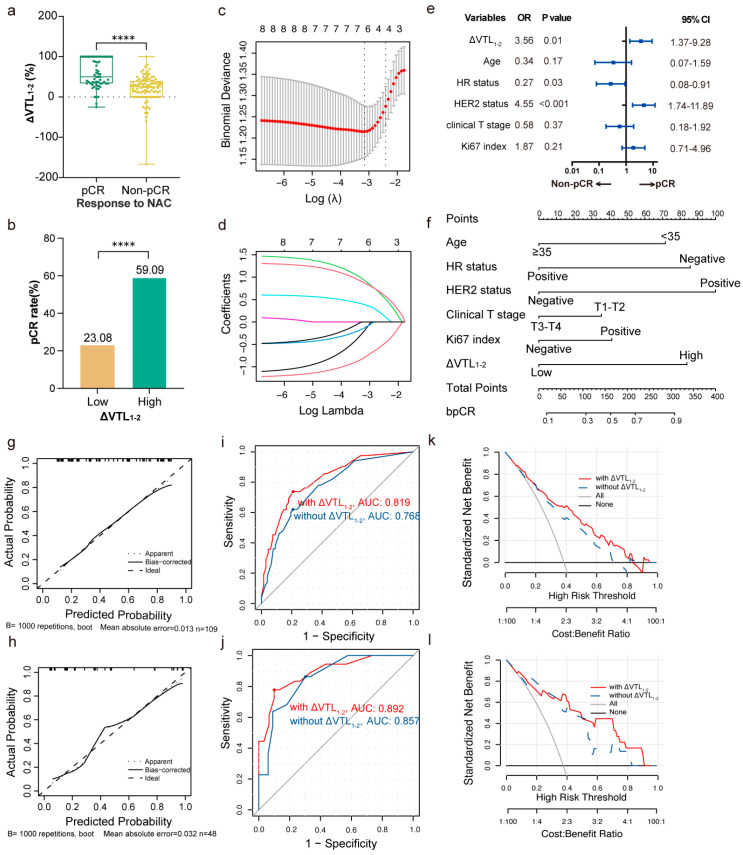
Feature selection, model development, and model validation for bpCR prediction. (**a**) ΔVTL_1-2_ between pCR and non-pCR groups (ΔVTL_1-2_ as a continuous variable). "****" indicates *p* < 0.0001, same with (**b**). (**b**) The pCR rates of patients with low-ΔVTL_1-2_ and high-ΔVTL_1-2_ (ΔVTL_1-2_ as a binary categorical variable). (**c**,**d**) Feature selection for predicting bpCR using LASSO-logistic regression. (**c**) LASSO algorithm using minimum penalty criteria from 10-fold cross-validation. ΔVTL_1-2_, age, hormone receptor status, HER2 status, clinical T stage, Ki-67 index, and BMI were extracted for model development. The red dots denote the mean deviances of the cross validation folds under different penalty λ. (**d**) LASSO coefficient profiles of candidate features. Each ldenotes the LASSO coefficient of a candidate feature. (**e**) Forest plot illustrating factors selected by LASSO regression in predicting bpCR using multivariate logistic regression. (**f**) Nomogram built for predicting bpCR based on multivariate logistic regression in the training set. (**g**) Calibration of the nomogram for the training set. (**h**) Calibration of the nomogram for the validation set. (**i**) Receiver operating characteristic curves (ROC) of different predictive models for the training set. (**j**) ROC of different predictive models for the validation set. (**k**) Decision curve analysis (DCA) of different predictive models for the training set. (**l**) DCA of different predictive models for the validation set.

**Figure 3:**
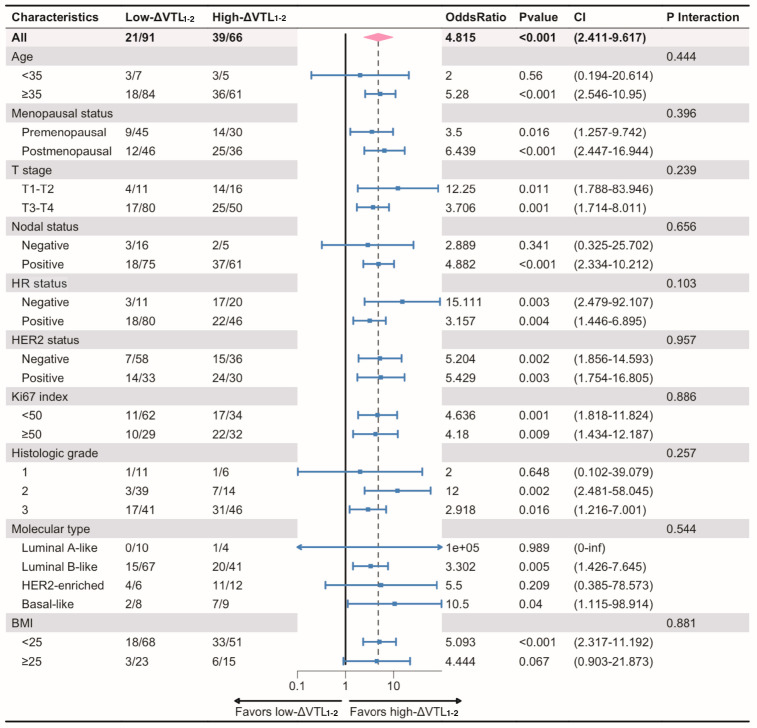
Subgroup analysis for bpCR according to ΔVTL_1-2_. Notes: ORs and 95% CIs were obtained from univariate logistic regression model. Interaction *p*-values were shown between ΔVTL_1-2_ and subgroups. Abbreviations: VTL, vessel through lesion; T, tumor; HR, hormone receptor; HER2, human epidermal growth factor receptor 2; BMI, body mass index; CI, confidential interval.

ΔVTL_1-2_ was higher in pCR group than in non-pCR group (*p* < 0.0001; Figure 2a). Meanwhile the pCR rate was higher in the high-ΔVTL_1-2_ group than in the low-ΔVTL_1-2_ group (*p* < 0.0001; Figure 2b). The multivariate logistic regression analysis suggested that ΔVTL_1-2_ served as an independent predictive factor for bpCR (OR 3.99; 95% CI 1.77–9.01; *p* < 0.001) (Supplementary Table S3). Subgroup analysis indicated that advantages in favor of high ΔVTL_1-2_ for bpCR across almost all subgroups. No interaction was detected between clinicopathological variables and ΔVTL_1-2_ for bpCR (Figure 3). Advantages in favor of high ΔVTL_1-3_ for bpCR were also detected across almost all subgroups (Supplementary Figure S1).

### 3.3. Model Development and Validation for bpCR

Eligible factors in predicting bpCR including ΔVTL_1-2_, age, HR status, HER2 status, clinical T stage, Ki-67 index, and BMI were selected with LASSO logistic regression in the training set (Figure 2c,d). ΔVTL_1-2_ was an independent predictor of bpCR (OR 3.56; 95% CI 1.37–9.28; *p* = 0.01), along with other clinicopathological factors (Figure 2e). A nomogram was created based on the multivariate model including the selected factors (Figure 2f). The accuracy of the model was evaluated using a calibration curve, respectively, in the training and validation set (Figure 2g,h).

ROC curves were created to compare the effectiveness of the different models with and without ΔVTL_1-2_. AUC of the model combining ΔVTL_1-2_ with clinicopathological factors for the training set was 0.819, while the model based on only clinicopathological factors reached an AUC of 0.768 (Figure 2i). For the validation set, AUCs of the two models were 0.892 and 0.857, respectively (Figure 2j). The DCA curve of the model with ΔVTL_1-2_ confirmed the superiority of the model with ΔVTL_1-2_ (Figure 2k,l).

High ΔVTL_1-3_ was also significantly associated with bpCR (Supplementary Figure S2A,B). A bpCR-predictive nomogram was also developed based on ΔVTL_1-3_ and seven other clinicopathological characteristics extracted by LASSO logistic regression in the training set (Supplementary Figure S2C–F). The model’s accuracy was evaluated using calibration curves (Supplementary Figure S2G,H). ROC curves compared the effectiveness of models with and without ΔVTL_1-3_, both indicating the superior predictive ability of the model with ΔVTL_1-3_ (Supplementary Figure S2I,J). DCA curves showed higher net benefit for the model with ΔVTL_1-3_ (Supplementary Figure S2K,L).

Additionally, we conducted exploratory analyses using ΔVTL_1-2_ and ΔVTL_1-3_ alone to build a predictive model for bpCR, compared with the model with only clinicopathological factors (including age, hormone receptor status, HER2 status, clinical T stage, Ki-67 index, BMI, and lymph node status). The MRI-only model achieved an AUC of 0.789 in the validation set, which was slightly lower than the clinicopathological model but still demonstrated favorable predictive capability (Supplementary Figure S3).

### 3.4. Model Development and Validation for RFS

**Figure 4:**
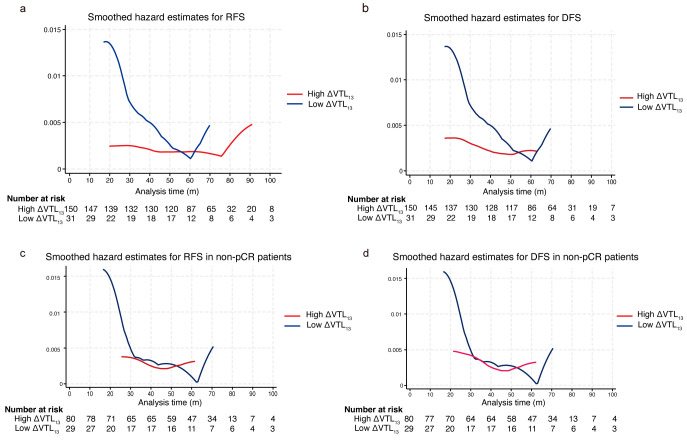
Time-dependent hazard rate by ΔVTL_1-3_ for RFS and DFS. (**a**) Smoothed hazard rate estimates for RFS. (**b**) Smoothed hazard rate estimates for DFS. (**c**) Smoothed hazard rate estimates for RFS in non-pCR patients. (**d**) Smoothed hazard rate estimates for DFS in non-pCR patients.

ΔVTL_1-3_ was shown to be an independent predictor of RFS according to the univariate (hazard ratio 0.28; 95% CI 0.14–0.57; *p* < 0.001) and multivariate cox regression analysis in the whole set (hazard ratio 0.23; 95% CI 0.11–0.50; *p* < 0.001) (Supplementary Table S4). The annual recurrence hazard curves of the different subgroups exhibited significant temporal heterogeneity. The low ΔVTL_1-3_ group exhibited a pronounced peak in recurrence and metastasis hazard within the first 2 years, with the peak occurring around the 20th month. In contrast, the high ΔVTL_1-3_ group showed a relatively steady and lower recurrence risk, with no noticeable peak observed within the 5-year period. Within the first two years post-operation, the incidence of RFS and DFS events was significantly higher in the low ΔVTL_1-3_ group compared to the high ΔVTL_1-3_ group (Figure 4a,b), which was particularly evident in the non-pCR population (Figure 4c,d).

**Figure 5:**
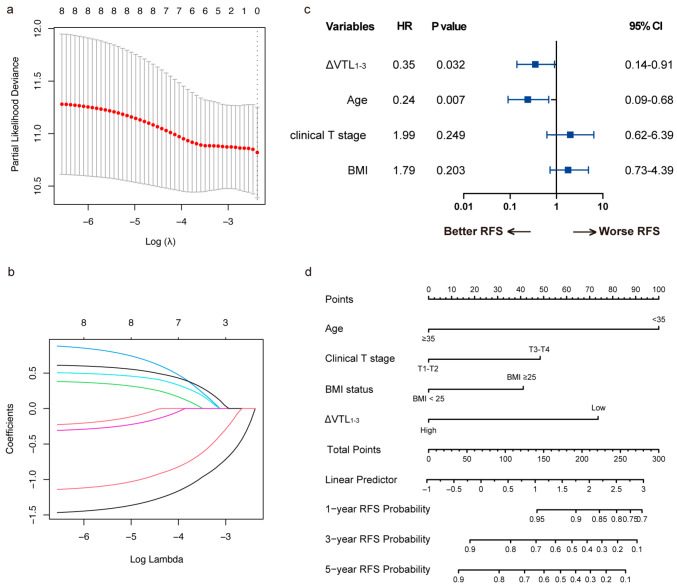
Feature selection and model development for RFS prediction. (**a**,**b**) Feature selection for predicting RFS using LASSO-cox regression. (**a**) LASSO algorithm using minimum penalty criteria from 10-fold cross-validation. ΔVTL_1-3_, age, clinical T stage, and BMI were extracted for model development. The red dots denote the mean deviances of the cross validation folds under different penalty λ. (**b**) LASSO coefficient profiles of candidate features. Each line denotes the LASSO coefficient of a candidate feature. (**c**) Forest plot illustrating factors selected by LASSO regression in predicting RFS using multivariate cox regression. (**d**) Nomogram built for predicting RFS based on multivariate cox regression.

**Figure 6:**
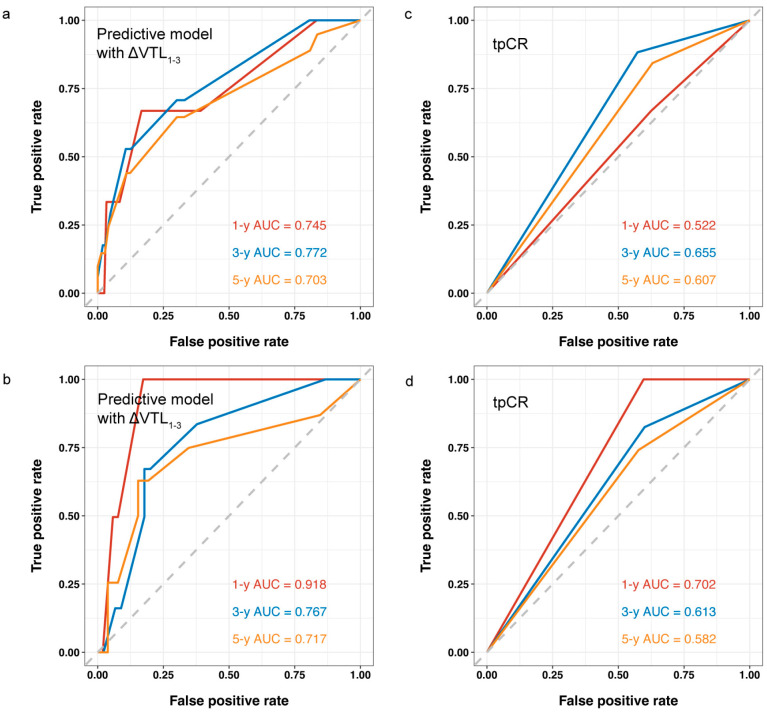
ROC analysis of different predictive models for RFS. (**a**) ROC of predictive model with ΔVTL_1-3_ for the training set. (**b**) ROC of predictive model with ΔVTL_1-3_ for the validation set. (**c**) ROC of tpCR alone for RFS prediction for the training set. (**d**) ROC of tpCR alone for RFS prediction for the validation set. Abbreviations: VTL, vessel through lesion; tpCR, total pathological complete response; AUC, area under curve; RFS, relapse-free survival.

With LASSO regression, ΔVTL_1-3_, age, clinical T stage, BMI were selected for valuable factors of RFS prediction (Figure 5a,b). We established a nomogram based on these factors (Figure 5c,d), and compared the predictive value of this model with that based on tpCR for RFS. As shown in Figure 6, the predictive model with ΔVTL_1-3_ revealed a stronger correlation with RFS in the training set (AUCs for 1-year, 3-year, and 5-year RFS of 0.745, 0.772, and 0.703, respectively, Figure 6a) and the validation set (AUCs for 1-year, 3-year, and 5-year RFS of 0.918, 0.767, and 0.717, respectively, Figure 6b), outperforming the model using tpCR in the training set (AUCs for 1-year, 3-year, and 5-year RFS of 0.522, 0.655, and 0.607, respectively, Figure 6c) and the validation set (AUCs for 1-year, 3-year, and 5-year RFS of 0.702, 0.613, and 0.582, respectively, Figure 6d).

The DCA curves indicated that using the predictive model with ΔVTL_1-3_ added more benefit than using tpCR in both sets (Supplementary Figure S4). The corresponding calibration curve showed promising agreement between the predicted RFS and the observed results in the training set (Supplementary Figure S5).

Compared with the clinicopathological model for RFS, the MRI-only model showed better performance, with AUCs of 0.944, 0.808, and 0.816 at 1, 3, and 5 years, respectively, in the validation set, which were higher than the corresponding AUCs of the clinicopathological model (Supplementary Figure S6).

### 3.5. Risk Stratification of the RFS Predictive Model

**Figure 7:**
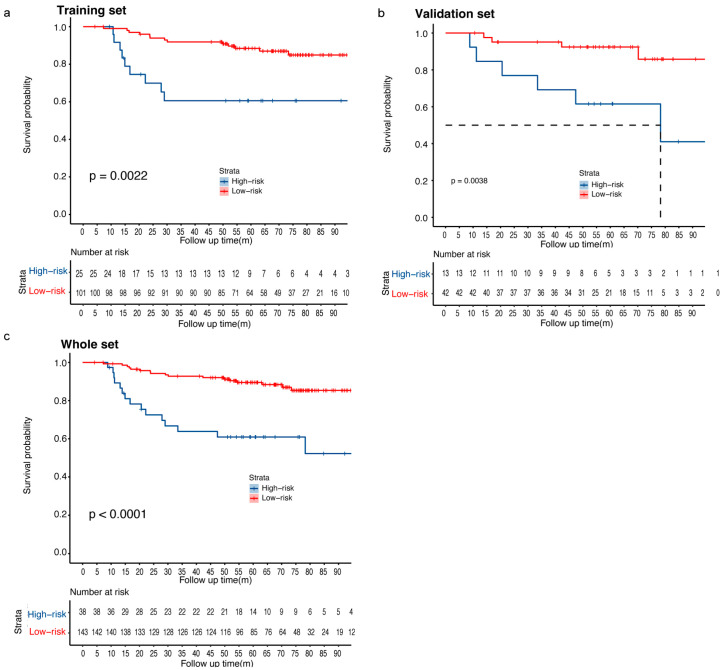
Kaplan–Meier survival curves of RFS according to the risk score predicted by the nomogram in (**a**) training set, (**b**) validation set, and (**c**) whole set.

Patients were stratified into two risk groups (high-risk vs. low-risk) according to the optimal cutoff of risk scores for RFS. The high-risk group showed poorer RFS than the low-risk group presented by Kaplan–Meier curves (Figure 7a–c; *p* = 0.0022 for the training set, *p* = 0.0038 for the validation set; *p* < 0.0001 for both of them combined). In the whole set, the 1-year, 3-year, and 5-year RFS rates were 89.3%, 63.9%, and 61.0%, respectively, for high-risk patients, versus 99.3%, 92.8%, and 89.5%, respectively, for low-risk patients. The Kaplan–Meier curves in various subgroups according to HR, HER2, and pCR status also showed good distinguishability of RFS prediction when stratified by risk groups (Supplementary Figure S7A–C). In order to evaluate the ability of the nomogram, the optimal cutoffs of risk score were applied for risk stratification for DFS and OS. The Kaplan–Meier curves for DFS and OS revealed favorable predictive value of the nomogram (Supplementary Figures S8 and S9).

## 4. Discussion

To the best of our knowledge, this study is the first to observe that dynamic evolution of vascular features can effectively predict bpCR. A predictive model based on changes in vascular features after two cycles of NAC could early forecast bpCR, exhibiting superior predictive performance compared to models relying on clinical-pathological factors. Additionally, our study is also the first to identify that the different dynamic evolution of vascular features post-NAC serves as an independent prognostic factor, which is associated with distinctly different temporal patterns of post-surgical recurrence and metastasis, and further clues a fundamental heterogeneity in tumor characteristics, especially apparent in non-pCR patients. Furthermore, a predictive model for prognosis was developed based on changes in vascular features during NAC, with predictive efficacy surpassing that based on tpCR.

Breast MRI angiography has become an integral part of standard breast examinations with the use of contrast-enhanced imaging. The typical MIP images generated from postprocessing subtracted images not only identify enhancing lesions but also present a vascular map of vessels within the breast [8,14,15]. This study aims to provide a more profound understanding of how vascular dynamics during NAC correlates with tumor characteristics, angiogenesis, and tumor microenvironment, offering potentially valuable insights for clinical practice. Angiogenesis, the formation of new blood vessels, is crucial for the growth and metastasis of solid tumors, which require a sustained blood supply for rapid proliferation [16]. This process is regulated by a complex interplay of proangiogenic factors, such as vascular endothelial growth factor (VEGF), which can also act directly on tumor cells, promoting tumor progression and creating a malignant positive feedback loop [17]. Bahhnassy et al. found that triple-negative breast cancer (TNBC) patients exhibit higher VEGF expression compared to non-TNBC patients, with elevated levels of VEGF-A in the blood associated with poor prognosis [18]. Furthermore, various malignancies, including esophageal cancer, colon cancer, and osteosarcoma, demonstrate different VEGF expression levels, which are correlated with tumor stage, molecular grade, and prognosis [19,20,21,22]. High VEGF expression is often linked to metastasis and poor outcomes [23,24]. The varying expression of angiogenesis-related factors suggests that tumor tissues differ in their sensitivity to treatment [25]. These changes can also be monitored throughout the course of treatment to assess therapeutic response. Kim et al. found that a decrease in VEGF transcripts is significantly associated with favorable treatment outcomes [26]. The aforementioned studies partially support our findings. The different dynamic changes in ΔVTL observed in this study might be related to corresponding fluctuation of angiogenic factors such as VEGF and further reflect the temporal heterogeneity of the tumor, at least partially representing a comprehensive manifestation of the tumor’s intrinsic characteristics.

Our study focused on predicting pathological response to NAC by calculating the relative change in the number of vessels compared to baseline (ΔVTL) and found that integrating ΔVTL_1-2_ with clinicopathological factors effectively predicts bpCR. Additionally, models constructed using ΔVTL_1-3_ also demonstrated good predictive performance. Bufi et al. found that patients with an asymmetric increase in breast vascularity in the affected breast had a higher likelihood of achieving pCR [10]. Similarly, Martincich et al. explored the relationship between changes in the number of large vessels in the affected breast and pathological response to NAC, observing a significant decrease in average vessel counts between responders and nonresponders (*p* = 0.015) [27]. Preclinical studies have also highlighted the anti-angiogenic activity of paclitaxel, contributing to its anti-tumor efficacy in vivo [28,29,30,31]. Lau et al. demonstrated that paclitaxel exhibits antiangiogenic activity by downregulating VEGF in a highly vascularized transgenic murine Met-1 breast cancer model, administered at non-cytotoxic doses, paclitaxel significantly reduced intratumoral angiogenesis and also suppressed VEGF expression in the tumors [32]. Studies have shown that paclitaxel can inhibit endothelial cell proliferation, chemotaxis, and morphogenesis in vitro—functions closely related to angiogenesis—and suppress angiogenesis itself in vivo using chick and mouse models [28,33]. Additionally, paclitaxel also downregulated VEGFR2 expression and upregulated thrombospondin-1 (TSP-1) expression in a metastatic mouse model [34]. All these studies were consistent with us, at least partially, and have provided evidence supporting that different dynamic change patterns of vessels during NAC, which can be detected early and conveniently through changes in ΔVTL, might be considered as a surrogate for tumor heterogeneity, and then further reflect sensitivities to treatment.

Through the study of the time distribution of recurrence risk in subgroups with different patterns of vascular changes, we observed for the first time that the low ΔVTL_1-3_ group experienced a rapid hazard peak in recurrence and metastasis, while the high ΔVTL_1-3_ group maintained consistently low recurrence levels without a noticeable peak. This disparity was particularly pronounced in the non-pCR subgroup. This might suggest that the differences in microenvironment and tumor heterogeneity between the two subgroups result in different reactivation times and growth patterns of dormant tumor cells in metastasis, displaying distinct recurrence patterns for the two subgroups. Gasparini et al.’s study showed that patients with a high angiogenesis index in their primary tumors have a higher risk of recurrence compared to patients with poorly vascularized tumors [35]. Moreover, patients with high intratumoral microvessel density (IMD) or elevated VEGF protein levels have significantly worse prognoses compared to those with low IMD or VEGF levels [36,37]. The aforementioned studies, either directly or indirectly, support our findings. Tumor dormancy has long been a clinical challenge, as dormant tumor cells can, in some cases, re-enter the proliferative phase and cause tumor recurrence [38,39]. The “angiogenic switch” has been confirmed as a critical step in tumor progression, marking the transition of the tumor from a dormant state to one of angiogenesis, which promotes tumor growth [40]. During this process, the balance between pro-angiogenic and anti-angiogenic factors is disrupted, favoring angiogenesis. Research suggests that this switch is regulated by various signals, such as VEGFA and TSP-1 [41]. In this study, the low ΔVTL_1-3_ subgroup showed no significant reduction in blood vessels, as tumor cells may have a strong ability to secrete angiogenic factors and be less affected by treatment. The micro-metastatic foci may maintain the attributes of the primary lesion and keep some abilities to actively secrete angiogenesis-related factors like VEGF, which can induce angiogenesis and stimulate early recurrence and metastasis post-operation. It seems reasonable to hypothesize that the heterogeneity in dynamic changes in vascular features during NAC reflects the tumor’s intrinsic traits and the nature of its micro-metastases. Low ΔVTL_1-3_ tumors are more prone to early recurrence, which is also observed in non-pCR subgroup, underscoring the value of tailoring follow-up strategies and treatment according to subgroups with different vascular change patterns. Especially in non-pCR patients, intensified treatment may be particularly significant for those with limited vascular reduction, although further research is needed to confirm this.

There are several limitations in this study. Firstly, it is a retrospective analysis. However, our patient selection was derived from a prospectively maintained database, it is exploratory and has been validated in both the training and validation sets. Therefore, this could indicate the existence of underlying intrinsic patterns to some degree, providing clues for future prospective research. Secondly, with a relatively small sample size, further validation in larger cohorts is expected. Thirdly there is an imbalance in the distribution of patients across different subtypes in the dataset, with a predominance of Luminal-type patients. Future studies should aim to expand the sample size, with a particular focus on triple-negative subtypes.

## 5. Conclusions

In conclusion, we first reported that the dynamic evolution of vascular features can effectively predict bpCR and that the different dynamic evolution of vascular features post-NAC serves as an independent prognostic factor, which is associated with distinctly different temporal post-surgical recurrence and metastasis patterns, especially apparent in non-pCR subgroup patients. It is a novel and practical direction of integration of dynamic alterations in vascular features with clinical-pathological parameters to formulate predictive models for the early anticipation of tumor pathological response and prognosis. These predictive instruments serve as invaluable tools for the identification of patients likely to benefit from NAC and better individualizing adjuvant treatment plans for patients after NAC, particularly in designing adjuvant chemotherapy and intensified adjuvant therapy for non-pCR patients.

## Figures and Tables

**Table 1:** Clinicopathological characteristics, MRI vascular features and outcome data of patients in the training and validation set.

Variable	Whole Set (n = 182)	Training Set (n = 127)	Validation Set (n = 55)	Statistic	*p*-Value
Age, n (%)				χ^2^ = 0.196	0.658
<35	14 (7.69)	11 (8.66)	3 (5.45)		
≥35	168 (92.31)	116 (91.34)	52 (94.55)		
Menopausal status, n (%)				χ^2^ = 0.052	0.819
Premenopausal	87 (47.8)	60 (47.24)	27 (49.09)		
Postmenopausal	95 (52.2)	67 (52.76)	28 (50.91)		
Clinical T stage, n (%)				χ^2^ = 0.580	0.446
T1–T2	36 (19.78)	27 (21.26)	9 (16.36)		
T3–T4	146 (80.22)	100 (78.74)	46 (83.64)		
Nodal status, n (%)				χ^2^ = 0.278	0.598
Negative	26 (14.29)	17 (13.39)	9 (16.36)		
Positive	156 (85.71)	110 (86.61)	46 (83.64)		
HER2 status, n (%)				χ^2^ = 0.829	0.363
Negative	110 (60.44)	74 (58.27)	36 (65.45)		
Positive	72 (39.56)	53 (41.73)	19 (34.55)		
Ki-67 index, n (%)				χ^2^ = 0.751	0.386
<50%	108 (59.34)	78 (61.42)	30 (54.55)		
≥50%	74 (40.66)	49 (38.58)	25 (45.45)		
Histologic grade, n (%)				χ^2^ = 2.481	0.289
G1	20 (10.99)	13 (10.24)	7 (12.73)		
G2	60 (32.97)	38 (29.92)	22 (40.00)		
G3	102 (56.04)	76 (59.84)	26 (47.27)		
Molecular subtype, n (%)				χ^2^ = 1.762	0.623
Luminal A-like	19 (10.44)	14 (11.02)	5 (9.09)		
Luminal B-like	122 (67.03)	82 (64.57)	40 (72.73)		
HER2-enriched	21 (11.54)	17 (13.39)	4 (7.27)		
Basal-like	20 (10.99)	14 (11.02)	6 (10.91)		
BMI, n (%)				χ^2^ = 0.023	0.881
<25	137 (75.27)	96 (75.59)	41 (74.55)		
≥25	45 (24.73)	31 (24.41)	14 (25.45)		
bpCR, n (%)				χ^2^ = 0.006	0.936
Non-pCR	110 (60.44)	77 (60.63)	33 (60.00)		
pCR	72 (39.56)	50 (39.37)	22 (40.00)		
ΔVTL_1-2_, %, Mean ± SD	34.50 ± 38.36	32.59 ± 39.08	38.83 ± 36.70	t = −0.938	0.349
ΔVTL_1-3_, %, Mean ± SD	53.72 ± 39.59	54.34 ± 40.89	52.28 ± 36.73	t = 0.322	0.748
ΔVTL_1-2_, n (%)				χ^2^ = 0.004	0.950
Low	91 (57.96)	63 (57.80)	28 (58.33)		
High	66 (42.04)	46 (42.20)	20 (41.67)		
ΔVTL_1-3_, n (%)				χ^2^ = 0.318	0.573
Low	32 (17.58)	21 (16.54)	11 (20.00)		
High	150 (82.42)	106 (83.46)	44 (80.00)		
RFS rate, % (95% CI)				t = 0.167	0.875
1-y	97.2 (94.8–99.6)	97.6 (94.9–100.0)	96.3 (91.5–100.0)		
3-y	86.8 (82.0–92.0)	86.0 (80.0–92.4)	88.7 (80.6–97.7)		
5-y	83.6 (78.3–89.4)	83.2 (76.7–90.2)	84.6 (75.3–95.0)		
OS rate, % (95% CI)				t = 0.472	0.661
1-y	100.0 (100.0–100.0)	100.0 (100.0–100.0)	100.0 (100.0–100.0)		
3-y	94.0 (90.6–97.5)	93.7 (89.6–98.0)	94.5 (88.7–100.0)		
5-y	91.7 (87.8–95.8)	90.6 (85.6–95.8)	94.5 (88.7–100.0)		

Note: All *p*-values adjusted for multiple comparisons using the Benjamini–Hochberg method (FDR) were >0.05. Abbreviations: T, tumor; ER, estrogen receptor; PR, progesterone receptor; HER2, human epidermal growth factor receptor 2; BMI, body mass index; bpCR, breast pathological complete response; HR, hormone receptor; VTL, vessel through lesion; RFS, relapse-free survival; OS, overall survival; CI, confidential interval.

**Table 2:** Relationships of ΔVTL_1-2_ and ΔVTL_1-3_ with clinicopathological characteristics.

Characteristics	ΔVTL_1-2_				ΔVTL_1-3_			
	Low, n = 91	High, n = 66	*p*-Value	Adjusted *p*-Value	Low, n = 32	High, n = 150	*p*-Value	Adjusted *p*-Value
Age(yr)			1	1			0.275	0.393
<35	7 (7.69%)	5 (7.58%)			4 (12.5%)	10 (6.67%)		
≥35	84 (92.3%)	61 (92.4%)			28 (87.5%)	140 (93.3%)		
Menopausal status			0.739	0.924			0.101	0.337
Premenopausal	45 (49.5%)	30 (45.5%)			20 (62.5%)	67 (44.7%)		
Postmenopausal	46 (50.5%)	36 (54.5%)			12 (37.5%)	83 (55.3%)		
BMI			0.858	0.953			1	1
<25	68 (74.7%)	51 (77.3%)			24 (75.0%)	113 (75.3%)		
≥25	23 (25.3%)	15 (22.7%)			8 (25.0%)	37 (24.7%)		
HR status			0.009	0.045			0.167	0.347
Negative	11 (12.1%)	20 (30.3%)			3 (9.38%)	33 (22.0%)		
Positive	80 (87.9%)	46 (69.7%)			29 (90.6%)	117 (78.0%)		
HER2 status			0.32	0.457			0.208	0.347
Negative	58 (63.7%)	36 (54.5%)			23 (71.9%)	87 (58.0%)		
Positive	33 (36.3%)	30 (45.5%)			9 (28.1%)	63 (42.0%)		
Ki-67 status			0.052	0.150			0.549	0.686
<50%	62 (68.1%)	34 (51.5%)			21 (65.6%)	87 (58.0%)		
≥50%	29 (31.9%)	32 (48.5%)			11 (34.4%)	63 (42.0%)		
Clinical T Stage			0.075	0.150			1	1
T1–2	11 (12.1%)	16 (24.2%)			6 (18.8%)	30 (20.0%)		
T3–4	80 (87.9%)	50 (75.8%)			26 (81.2%)	120 (80.0%)		
Nodal status			0.114	0.190			0.175	0.347
Negative	16 (17.6%)	5 (7.58%)			7 (21.9%)	19 (12.7%)		
Positive	75 (82.4%)	61 (92.4%)			25 (78.1%)	131 (87.3%)		
Molecular type			0.069	0.150			0.056	0.280
Luminal A-like	10 (11.0%)	4 (6.06%)			7 (21.9%)	12 (8.00%)		
Luminal B-like	67 (73.6%)	41 (62.1%)			22 (68.8%)	100 (66.7%)		
HER2-enriched	6 (6.59%)	12 (18.2%)			1 (3.12%)	20 (13.3%)		
Basel-like	8 (8.79%)	9 (13.6%)			2 (6.25%)	18 (12.0%)		
Histologic grade			0.007	0.045			0.001	0.010
G1	11 (12.1%)	6 (9.09%)			6 (18.8%)	14 (9.33%)		
G2	39 (42.9%)	14 (21.2%)			17 (53.1%)	43 (28.7%)		
G3	41 (45.1%)	46 (69.7%)			9 (28.1%)	93 (62.0%)		

Note: *p*-values adjusted by Benjamini–Hochberg method (FDR control). Abbreviations: VTL, vessel through lesion; BMI, body mass index; HR, hormone receptor; HER2, human epidermal growth factor receptor 2; T, tumor.

## Data Availability

Data available on request due to privacy and ethical restrictions.

## Supplementary Materials

The following supporting information can be downloaded at: https://www.mdpi.com/article/10.3390/curroncol32060350/s1, Figure S1: Subgroup analysis for bpCR according to ΔVTL_1-3_; Figure S2: Feature selection, model development and model validation for bpCR prediction; Figure S3: ROC analysis of MRI-only model and clinicopathological model for bpCR; Figure S4: Decision curve analysis of 1-year, 3-year, 5-year RFS for predictive model with ΔVTL_1-3_ vs tpCR alone; Figure S5: Calibration curve for based on nomogram and tpCR alone in the training set; Figure S6: ROC analysis of MRI-only model and clinicopathological model for RFS; Figure S7: Kaplan–Meier survival curves of RFS according to the risk score predicted by the nomogram in subgroups; Figure S8: Kaplan–Meier survival curves of DFS according to the risk score predicted by the nomogram; Figure S9: Kaplan–Meier survival curves of OS according to the risk score predicted by the nomogram; Table S1: Relationships of VTL_1_, VTL_2_ and VTL_3_ with clinicopathological characteristics; Table S2. Relationships of ΔVTL_1-2_ and ΔVTL_1-3_ with clinicopathological characteristics; Table S3: Univariate and multivariate analysis for predictive factors of bpCR in the whole set; Table S4: Univariate and multivariate analysis for predictive factors of RFS in the whole set.

## Abbreviations

The following abbreviations are used in this manuscript:

AUC	area under the curve
bpCR	breast pathological complete response
DCA	decision curve analysis
DFS	disease-free survival
ER	estrogen receptor
HER2	human epidermal growth factor receptor-2
HR	hormone receptor
LABC	locally advanced breast cancer
LASSO	least absolute shrinkage and selection operator
MIP	maximum intensity projection
MRI	magnetic resonance imaging
NAC	neoadjuvant chemotherapy
OS	overall survival
PR	progesterone receptor
RFS	relapse-free survival
ROC	receiver operating characteristic
TNBC	triple-negative breast cancer
tpCR	total pathological complete response
TSP-1	thrombospondin-1
VEGF	vascular endothelial growth factor
VTL	vessel through lesion
