# Supplementary material for: Dynamic Evolution of Vascular Features Based on Magnetic Resonance Imaging to Predict Pathological Response, Patterns of Recurrence and Survival Outcomes in Breast Cancer Neoadjuvant Chemotherapy
Source: Curr Oncol. 2025 Jun 13;32(6):350. doi: 10.3390/curroncol32060350 (PMC12192275; doi:10.3390/curroncol32060350)
Supplement: Supplementary file 1 [file curroncol-32-00350-s001.zip › Supplementary Material Summary.pdf]

Supplementary Table S1. Relationships of VTL$_1$, VTL$_2$ and VTL$_3$ with clinicopathological characteristics.

Characteristics	VTL$_1$			VTL$_2$			VTL$_3$		
	**Low, n=108**	**High, n=74**	**P value**	**Low, n=78**	**High, n=79**	**P value**	**Low, n=114**	**High, n=68**	**P value**
Age			0.500			1.000			1.000
<35	10 (9%)	4 (5%)		6 (8%)	6 (8%)		9 (8%)	5 (7%)	
≥35	98 (91%)	70 (95%)		72 (92%)	73 (92%)		105 (92%)	63 (93%)	
Menopausal status			1.000			0.301			1.000
Premenopausal	52 (48%)	35 (47%)		41 (53%)	34 (43%)		54 (47%)	33 (49%)	
Postmenopausal	56 (52%)	39 (53%)		37 (47%)	45 (57%)		60 (53%)	35 (51%)	
BMI			0.263			0.045			0.096
<25	85 (79%)	52 (70%)		65 (83%)	54 (68%)		91 (80%)	46 (68%)	
≥25	23 (21%)	22 (30%)		13 (17%)	25 (32%)		23 (20%)	22 (32%)	
HR status			0.480			0.041			0.256
Negative	19 (18%)	17 (23%)		21 (27%)	10 (13%)		26 (23%)	10 (15%)	
Positive	89 (82%)	57 (77%)		57 (73%)	69 (87%)		88 (77%)	58 (85%)	
HER2 status			0.320			0.171			0.045
Negative	69 (64%)	41 (55%)		42 (54%)	52 (66%)		62 (54%)	48 (71%)	
Positive	39 (36%)	33 (45%)		36 (46%)	27 (34%)		52 (46%)	20 (29%)	
Ki-67 status			0.626			0.170			0.326
<50%	62 (57%)	46 (62%)		43 (55%)	53 (67%)		64 (56%)	44 (65%)	
≥50%	46 (43%)	28 (38%)		35 (45%)	26 (33%)		50 (44%)	24 (35%)	
Clinical T Stage			0.001			<0.001			0.129
T1-2	31 (29%)	5 (7%)		23 (30%)	4 (5%)		27 (24%)	9 (13%)	
T3-4	77 (71%)	69 (93%)		55 (70%)	75 (95%)		87 (76%)	59 (87%)	
Nodal status			0.689			0.365			0.011
Negative	14 (13%)	12 (16%)		8 (10%)	13 (16%)		10 (9%)	16 (24%)	
Positive	94 (87%)	62 (84%)		70 (90%)	66 (84%)		104 (91%)	52 (76%)	
Molecular type			0.912			0.042			0.005
Luminal A-like	11 (10%)	8 (11%)		3 (4%)	11 (14%)		5 (5%)	14 (21%)	
Luminal B-like	74 (69%)	48 (65%)		53 (68%)	55 (70%)		79 (69%)	43 (63%)	
HER2-enriched	11 (10%)	10 (13%)		13 (17%)	5 (6%)		16 (14%)	5 (7%)	
Basel-like	12 (11%)	8 (11%)		9 (11%)	8 (10%)		14 (12%)	6 (9%)	
Histologic grade			0.102			0.333			0.019
G1	12 (11%)	8 (11%)		6 (8%)	11 (14%)		10 (9%)	10 (14%)	
G2	42 (39%)	18 (24%)		25 (32%)	28 (35%)		31 (27%)	29 (43%)	
G3	54 (50%)	48 (65%)		47 (60%)	40 (51%)		73 (64%)	29 (43%)	

Supplementary Table S2. Relationships of ΔVTL_{1-2} and ΔVTL_{1-3} with clinicopathological characteristics.

Characteristics	ΔVTL_{1-2}			ΔVTL_{1-3}		
	**Spearman r**	**95% CI**	***p*- value**	**Spearman r**	**95% CI**	***p*- value**
Ki-67 status	0.183	0.0222 - 0.334	0.020	0.254	0.108 - 0.388	<0.001
BMI	0.063	-0.0992 - 0.222	0.430	0.040	-0.110 - 0.189	0.59

Supplementary Table S3. Univariate and multivariate analysis for predictive factors of bpCR in the whole set.

Variables	Comparison for OR	Univariate analysis			Multivariate analysis		
		OR	95% CI	P	OR	95% CI	P
$\Delta VTL_{1\text{-}2}$	Low vs High	4.81	2.41-9.62	<0.001	3.99	1.77-9.01	<0.001
Age	<35 vs ≥35	0.63	0.21-1.88	0.41			
Menopause	Premenopausal vs Postmenopausal	1.65	0.91-3.02	0.1			
Clinical T stage	T1-2 vs T3-4	0.29	0.13-0.61	<0.001	0.35	0.12-0.98	0.05
Nodal Status	Negative vs Positive	1.94	0.77-4.88	0.16			
HR status	Negative vs Positive	0.29	0.13-0.61	<0.001	0.34	0.13-0.95	0.04
HER2 status	Negative vs Positive	4.6	2.43-8.72	<0.001	5.49	2.42-12.46	<0.001
Ki67 index	<50% vs ≥50%	3.09	1.66-5.73	<0.001	2.24	0.99-5.08	0.05
BMI	<25 vs ≥25	0.4	0.19-0.86	0.02	0.59	0.21-1.63	0.31

Abbreviations: VTL, vessel through lesion; T, tumor; HR, hormone receptor; HER2, human epidermal growth factor receptor 2; BMI, body mass index; OR, odds ratio; CI, confidential interval.

Supplementary Table S4. Univariate and multivariate analysis for predictive factors of RFS in the whole set.

Variables	Comparison for HR	Univariate analysis			Multivariate analysis		
		**HR**	**95% CI**	**P**	**HR**	**95% CI**	**P**
$\Delta VTL_{1\text{-}3}$	Low vs High	0.28	0.14-0.57	<0.001	0.23	0.11- 0.50	<0.001
Age	<35 vs ≥35	0.42	0.16-1.08	0.072	0.46	0.16- 1.31	0.146
Menopause	Premenopausal vs Postmenopausal	1.49	0.74-2.99	0.267			
T stage	T1-2 vs T3-4	1.14	0.49-2.67	0.757	1.22	0.50- 3.02	0.660
Nodal Status	Negative vs Positive	0.74	0.31-1.8	0.508	0.96	0.38-2.42	0.936
HR status	Negative vs Positive	0.9	0.39-2.09	0.804	0.63	0.25- 1.58	0.325
HER2 status	Negative vs Positive	0.96	0.48-1.92	0.899	1.05	0.50-2.20	0.901
Ki67 index	<50% vs ≥50%	1.49	0.75-2.95	0.255	1.54	0.74- 3.20	0.251
BMI	<25 vs ≥25	1.89	0.93-3.84	0.079	2.30	1.10- 4.82	0.027

Abbreviations: VTL, vessel through lesion; T, tumor; HR, hormone receptor; HER2, human epidermal growth factor receptor 2; BMI, body mass index; HR, hazard ratio; CI, confidential interval.

Supplementary Figure S1. Subgroup analysis for bpCR according to ΔVTL1-3.

Characteristics	Low-ΔVTL1-3	High-ΔVTL1-3	OddsRatio	Pvalue	CI	P Interaction
**All**	**2/32**	**70/150**	**13.125**	**0.001**	**(3.027-56.904)**	
Age						0.343
<35	1/4	6/10	4.5	0.256	(0.337-60.151)	
≥35	1/28	64/140	22.737	0.002	(3.009-171.807)	
Menopausal status						0.103
Premenopausal	2/20	27/67	6.075	0.022	(1.302-28.345)	
Postmenopausal	0/12	43/83	1e+05	0.988	(0-inf)	
T stage						0.057
T1-T2	2/6	21/30	4.667	0.106	(0.72-30.23)	
T3-T4	0/26	49/120	1e+05	0.989	(0-inf)	
Nodal status						0.199
Negative	1/7	6/19	2.769	0.391	(0.27-28.392)	
Positive	1/25	64/131	22.925	0.002	(3.014-174.378)	
HR status						0.284
Negative	0/3	23/33	1e+05	0.984	(0-inf)	
Positive	2/29	47/117	9.064	0.004	(2.057-39.943)	
HER2 status						0.147
Negative	0/23	28/87	1e+05	0.984	(0-inf)	
Positive	2/9	42/63	7	0.021	(1.336-36.686)	
Ki67 index						0.143
<50	0/21	31/87	1e+05	0.984	(0-inf)	
≥50	2/11	39/63	7.312	0.016	(1.455-36.742)	
Histologic grade						0.34
1	0/6	4/14	1e+05	0.992	(0-inf)	
2	0/17	11/43	1e+05	0.986	(0-inf)	
3	2/9	55/93	5.066	0.05	(0.998-25.725)	
Molecular type						0.662
Luminal A-like	0/7	1/12	1e+05	0.992	(0-inf)	
Luminal B-like	2/22	42/100	7.241	0.01	(1.605-32.676)	
HER2-enriched	0/1	17/20	1e+05	0.996	(0-inf)	
Basal-like	0/2	10/18	1e+05	0.995	(0-inf)	
BMI						0.157
<25	1/24	60/113	26.038	0.002	(3.401-199.352)	
≥25	1/8	10/37	2.593	0.4	(0.282-23.808)	

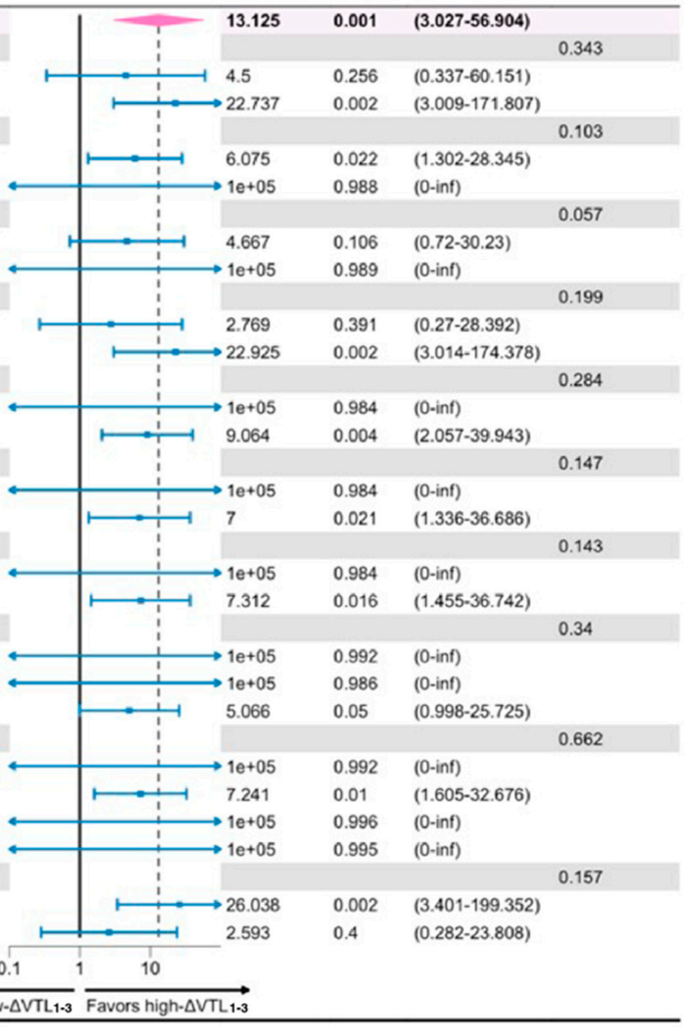

Notes: ORs and 95% CIs were obtained from univariate logistic regression model. Interaction P values were shown between ΔVTL1-3 and subgroups.

Abbreviations: VTL, vessel through lesion; T, tumor; HR, hormone receptor; HER2, human epidermal growth factor receptor 2; BMI, body mass index; CI, confidential interval.

Supplementary Figure S2. Feature selection, model development and model validation for bpCR prediction.

a $\Delta VTL_{1\text{-}3}$ between pCR and non-pCR groups.

b The pCR rates of patients with low-$\Delta VTL_{1\text{-}3}$ and high-$\Delta VTL_{1\text{-}3}$.

c, d Feature selection for predicting bpCR using LASSO-logistic regression.

c LASSO algorithm using minimum penalty criteria from10-fold cross-validation. $\Delta VTL_{1\text{-}3}$, Age, Hormone receptor status, HER2 status, clinical T stage, Ki-67 index, BMI and nodal status were extracted for model development.

d LASSO coefficient profiles of candidate features.

e Forest plot illustrating factors selected by LASSO regression in predicting bpCR using multivariate logistic regression.

f Nomogram built for predicting bpCR based on multivariate logistic regression in the training set.

g Calibration of the nomogram for the training set.

h Calibration of the nomogram for the validation set.

i Receiver operating characteristic curves (ROC) of different predictive models for the training set.

j ROC of different predictive models for the validation set.

k Decision curve analysis (DCA) of different predictive models for the training set.

l DCA of different predictive models for the validation set.

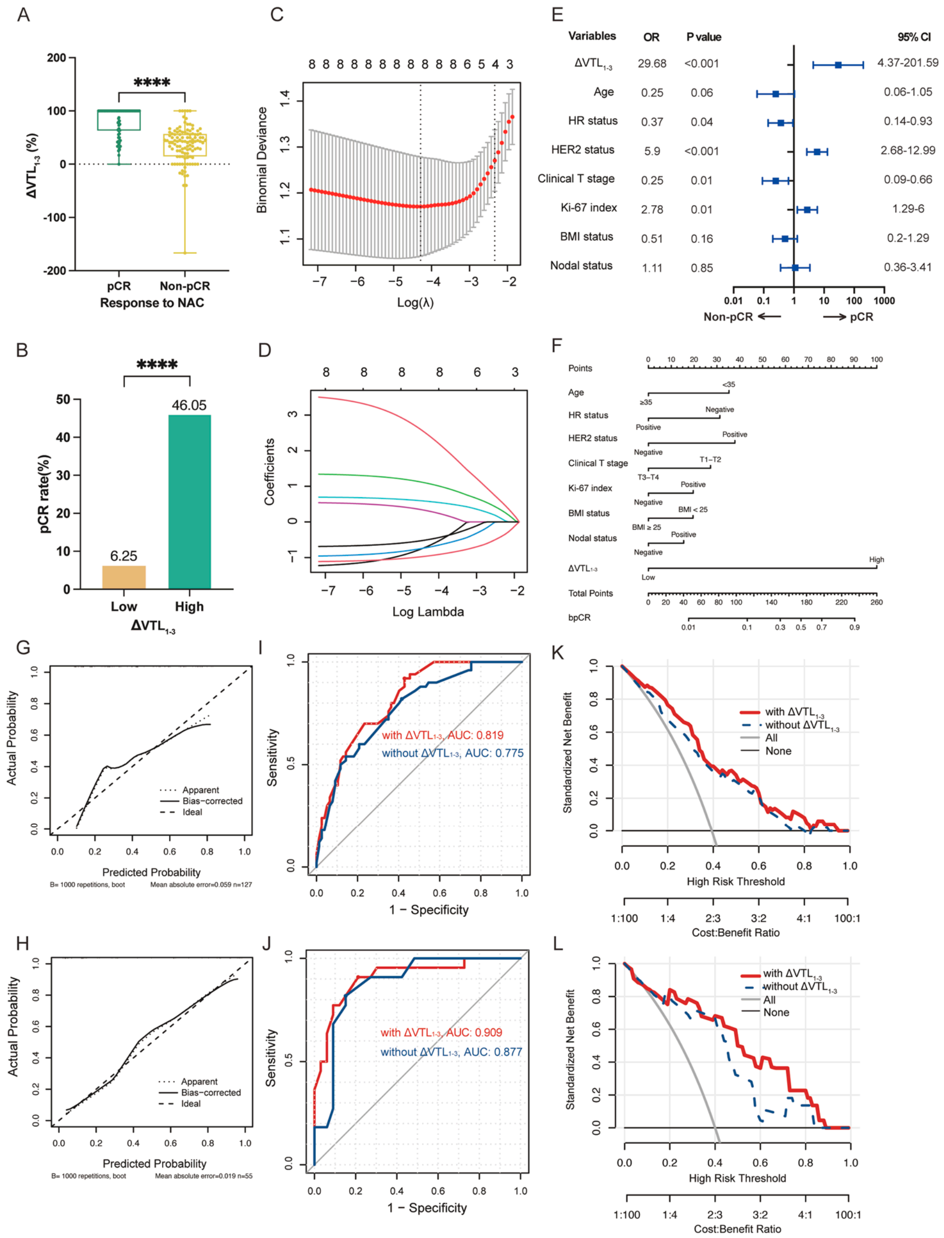

Abbreviations: bpCR, breast pathological complete response; VTL, vessel through lesion; HR, hormone receptor; HER2, human epidermal growth factor receptor 2; T, tumor; BMI, body mass index; OR, odds ratio; CI, confidential interval.

Supplementary Figure S3. ROC analysis of MRI-only model and clinicopathological model for bpCR.

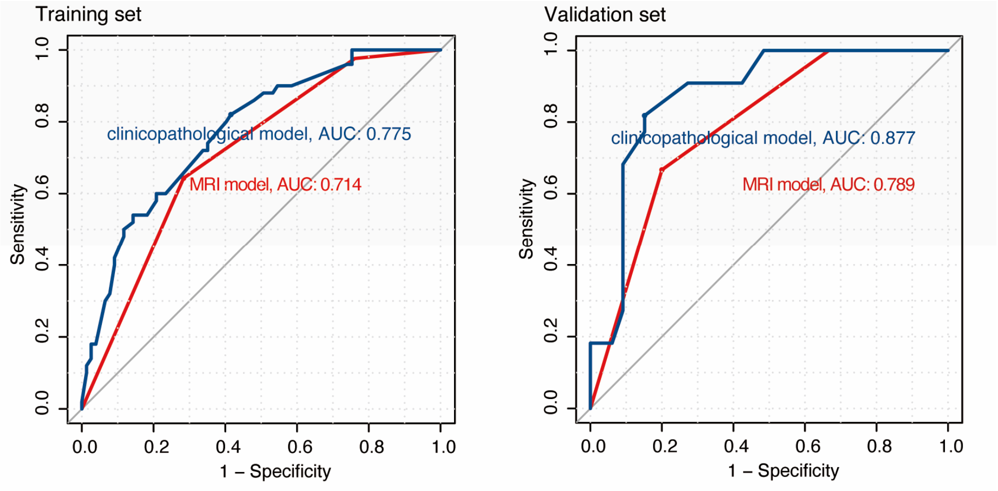

Supplementary Figure S4. Decision curve analysis of 1-year, 3-year, 5-year RFS for predictive model with ΔVTL_{1-3} vs tpCR alone in the (a) training and (b) validation set.

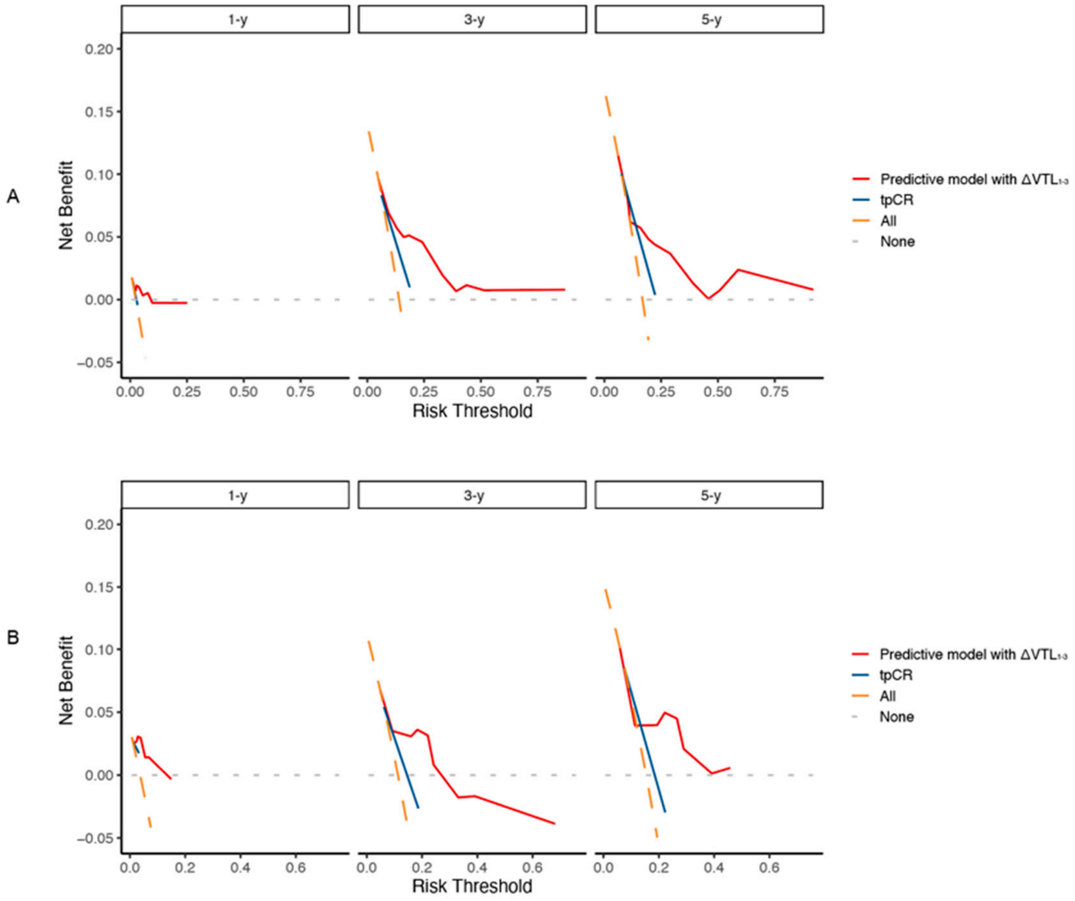

Abbreviations: VTL, vessel through lesion; tpCR, total pathological complete response; RFS, relapse-free survival.

Supplementary Figure S5. Calibration curve for (a) 1-year, (b) 3-year, (c) 5-year RFS prediction based on nomogram and tpCR alone in the training set.

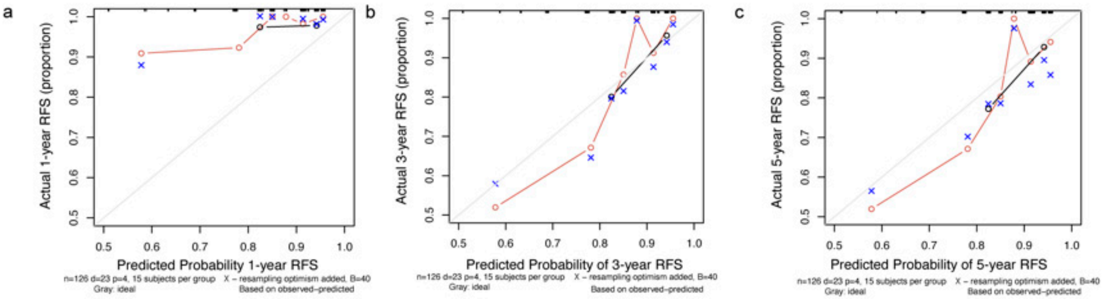

Abbreviations: RFS, relapse-free survival.

Supplementary Figure S6. ROC analysis of MRI-only model and clinicopathological model for RFS.

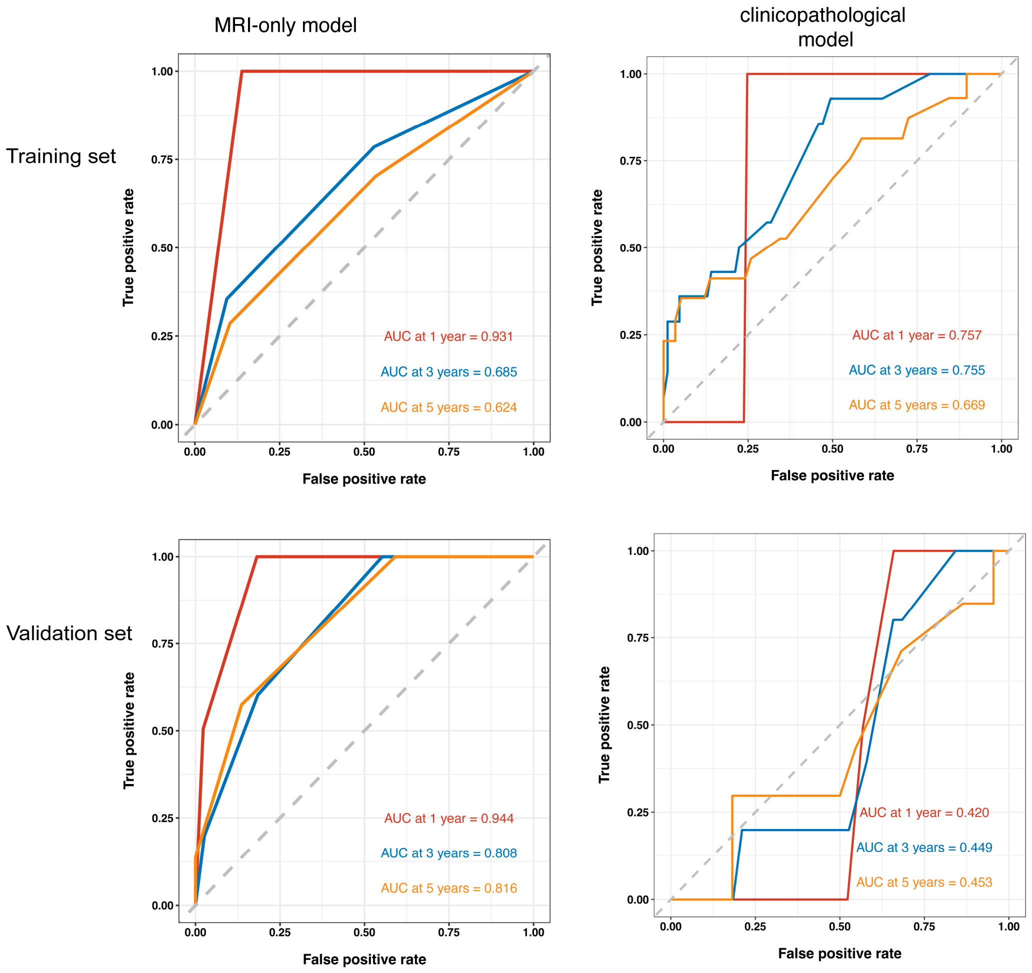

Supplementary Figure S7. Kaplan–Meier survival curves of RFS according to the risk score predicted by the nomogram in (a) HR-negative and HR-positive subgroups, (b) HER2-negative and HER2-positive subgroups, and (c) non-pCR and pCR subgroups.

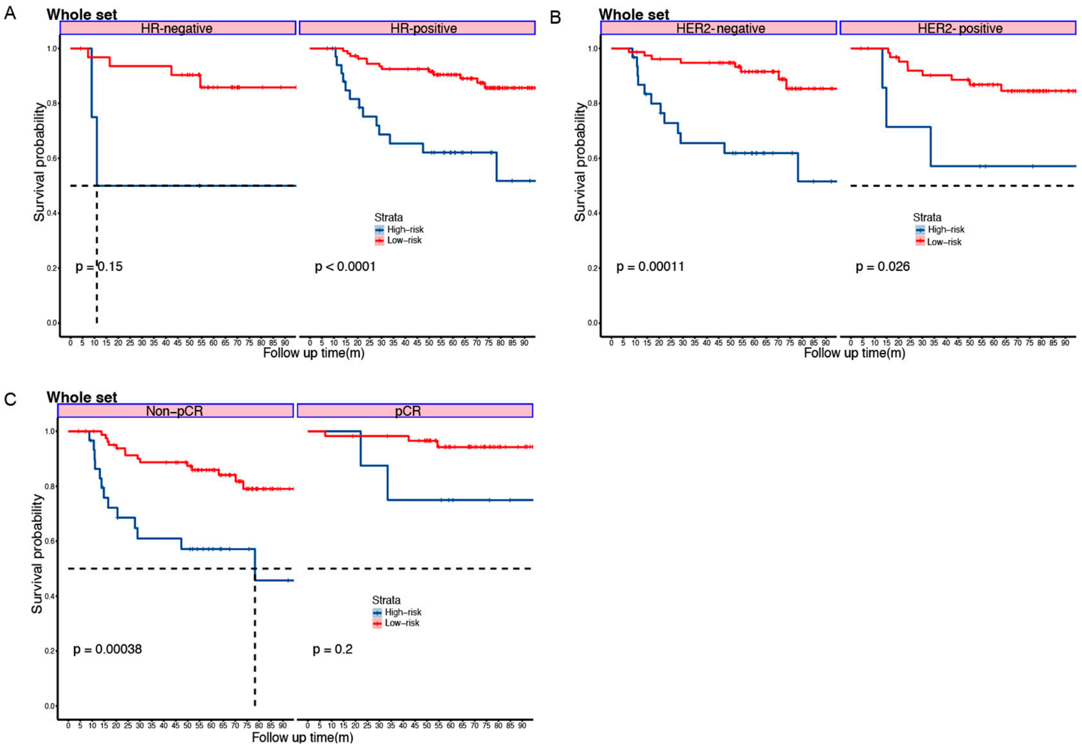

Abbreviations: HR, hormone receptor; HER2, human epidermal growth factor receptor 2; pCR, pathological complete response; RFS, relapse-free survival.

Supplementary Figure S8. Kaplan–Meier survival curves of DFS according to the risk score predicted by the nomogram in the (a) training set, (b) validation set, and (c) the whole set.

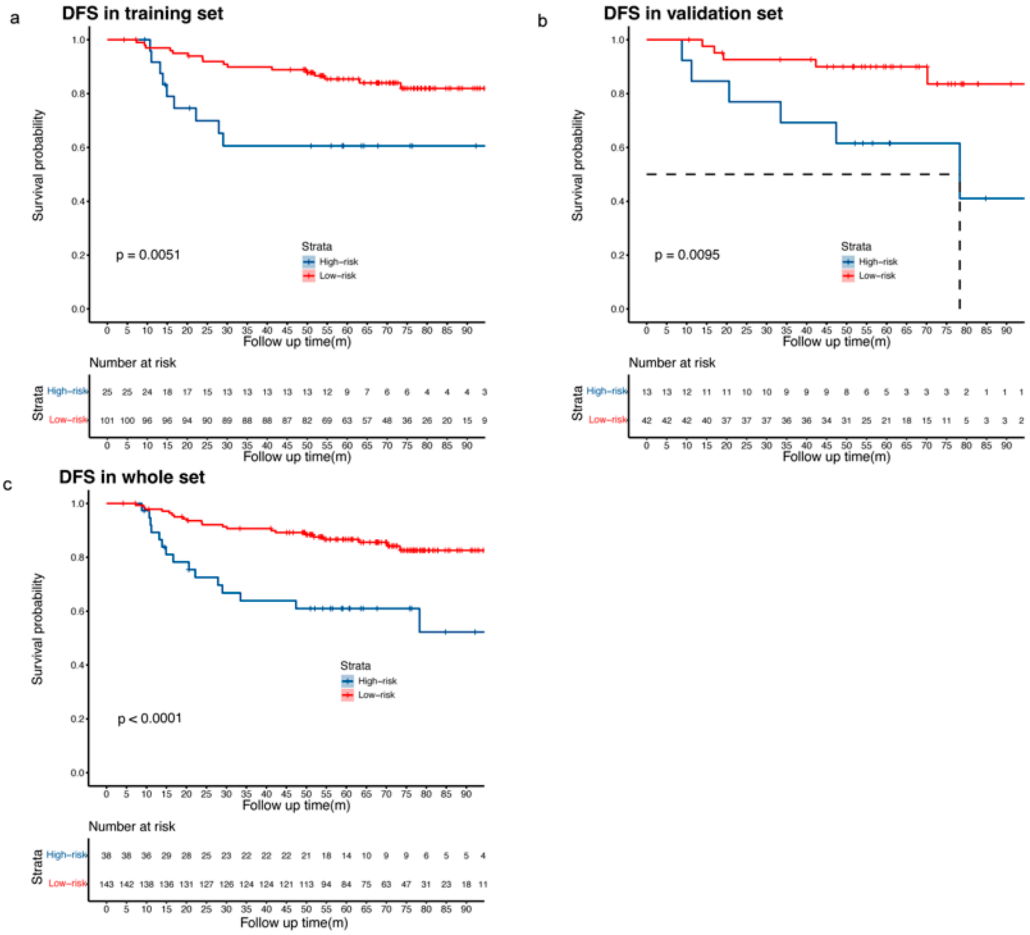

Abbreviations: DFS, disease free survival.

Supplementary Figure S9. Kaplan–Meier survival curves of OS according to the risk score predicted by the nomogram in the (a) training set, (b) validation set, and (c) the whole set.

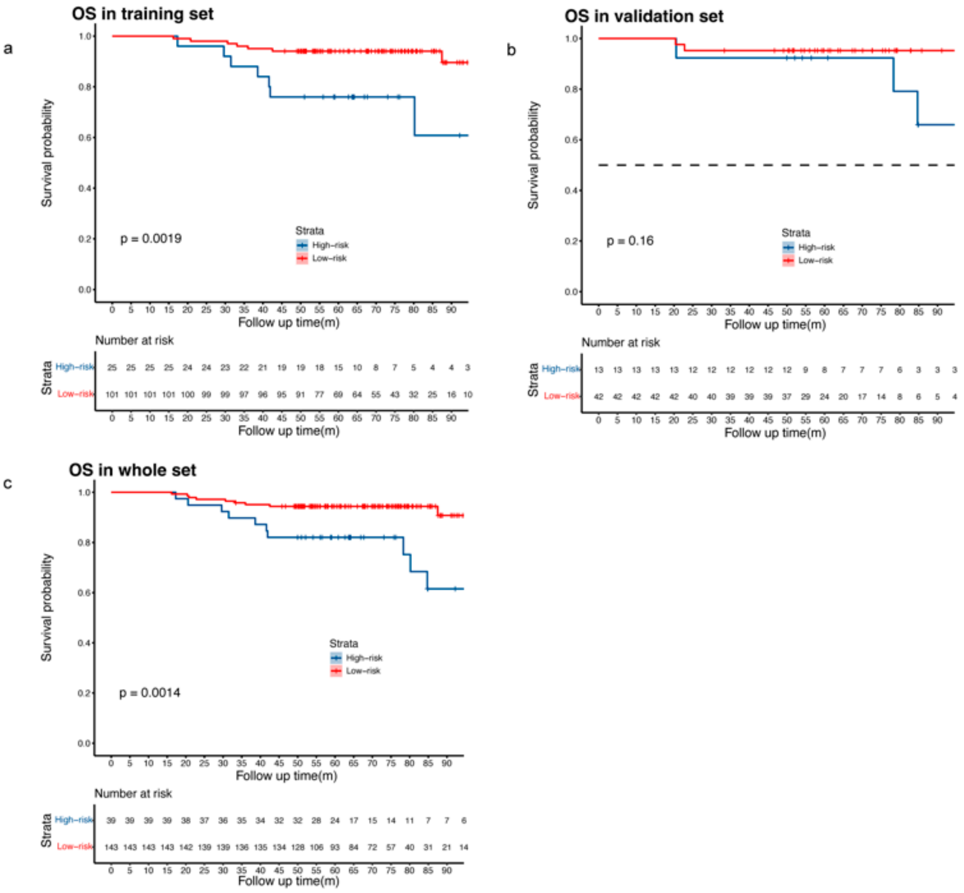

Abbreviations: OS, overall survival.
